# Characterization of Distinct Chondrogenic Cell Populations of Patients Suffering from Microtia Using Single-Cell Micro-Raman Spectroscopy

**DOI:** 10.3390/biomedicines11092588

**Published:** 2023-09-21

**Authors:** Dominika Zielinska, Hesham K. Yosef, Tilo Zollitsch, Johann Kern, Yvonne Jakob, David Gvaramia, Nicole Rotter, Luca Pontiggia, Ueli Moehrlen, Thomas Biedermann, Agnes S. Klar

**Affiliations:** 1 Tissue Biology Research Unit, University Children’s Hospital Zurich, 8952 Schlieren, Switzerland; 2 Children’s Research Center, University Children’s Hospital Zurich, 8032 Zurich, Switzerland; 3 Faculty of Medicine, University of Zurich, 8032 Zurich, Switzerland; 4 microphotonXGmbH, 82327 Tutzing, Germany; 5 Department of Otorhinolaryngology, Head and Neck Surgery, Medical Faculty Mannheim, University of Heidelberg, 68167 Mannheim, Germany; 6 Department of Surgery, University Children’s Hospital Zurich, 8032 Zurich, Switzerland

**Keywords:** Raman spectroscopy, chondrocytes, perichondrocytes, microtia

## Abstract

Microtia is a congenital condition of abnormal development of the outer ear. Tissue engineering of the ear is an alternative treatment option for microtia patients. However, for this approach, the identification of high regenerative cartilage progenitor cells is of vital importance. Raman analysis provides a novel, non-invasive, label-free diagnostic tool to detect distinctive biochemical features of single cells or tissues. Using micro-Raman spectroscopy, we were able to distinguish and characterize the particular molecular fingerprints of differentiated chondrocytes and perichondrocytes and their respective progenitors isolated from healthy individuals and microtia patients. We found that microtia chondrocytes exhibited lower lipid concentrations in comparison to healthy cells, thus indicating the importance of fat storage. Moreover, we suggest that collagen is a useful biomarker for distinguishing between populations obtained from the cartilage and perichondrium because of the higher spectral contributions of collagen in the chondrocytes compared to perichondrocytes from healthy individuals and microtia patients. Our results represent a contribution to the identification of cell markers that may allow the selection of specific cell populations for cartilage tissue engineering. Moreover, the observed differences between microtia and healthy cells are essential for gaining better knowledge of the cause of microtia. It can be useful for designing novel treatment options based on further investigations of the discovered biochemical substrate alterations.

## 1. Introduction

Microtia is a congenital disorder manifested by an external ear malformation or a lack of the entire external ear auricle (anotia) [1]. However, treatment methods are limited [2]. There are several hypotheses about the etiology of the underdevelopment of the auricle in microtia patients, yet the genetic and biochemical mechanisms underlying microtia auricle morphogenesis are not fully established. Existing data suggest that microtia is caused by inherited mutations or, in sporadic cases, by multifactorial variables. Importantly, animal studies indicate that the Homeobox gene 1 (*HOX1*) mutation is associated with an underdeveloped external ear [3,4].

To regenerate cartilage, it is essential to define progenitor cells by understanding the unique differences between them and the differentiated cells. Regeneration of mature cartilage is thought to be driven by chondrogenic progenitors in the perichondrium, which covers most non-articular cartilage [5]. It has been previously reported that transplanted perichondrium can form hyaline cartilage in vivo [6]. Therefore, defining a progenitor cell population by understanding their unique biochemical characteristics could improve the selection of chondroblasts from cartilage or perichondrium for cartilage regeneration. In addition, isolating chondroblasts from the perichondrium reduces the need for cartilage biopsies and, therefore, reduces the invasiveness of the procedure. In fact, the perichondrial donor site can spontaneously heal [7] due to nourishment from capillaries [8]. However, the molecular cell signatures of the different chondrocytes and perichondrocytes populations remain elusive.

Due to its high sensitivity, micro-Raman spectroscopy provides a non-invasive, label-free diagnostic tool for detecting distinctive molecular and biochemical features of single cells and/or tissues [9]. The Raman phenomenon was discovered by Sir C. V. Raman. It is a secondary radiation emerging after molecular vibrations induced by the interaction of light with matter [9]. Raman scattered photons emerging from this interaction are shifted in wavelength from that of the original excitation light. This Raman shift depends on the chemical structure of the excited molecule. Therefore, scattered Raman photons can reveal the chemical composition of the analyzed specimen and are considered to be a molecular fingerprint. A combination of micro-Raman and optical trapping enables non-invasive single-cell analysis. Implementing such a combination eliminates the need for cytospinning attachment of the cells to the substrate surface and allows trapping cells floating in liquids to accurately achieve the Raman measurement. It is increasingly becoming used more and more in the analysis of human tissues and cells [10]. For example, in clinical research, micro-Raman spectroscopy helps to distinguish healthy versus diseased cells in the diagnosis of breast cancer [11], lung cancer [12,13], cervical cancers [14], prostate cancer [15], and tumors of the oral mucosa [16]. Micro-Raman spectroscopy has also been applied as a tool in the analysis of bones [17], nails, or cartilage and is especially important for forensics and tissue engineering. One of the first significant achievements of Raman spectroscopy was the characterization of Raman spectra for collagen, elastin, and gelatin [18], which is essential for the analysis of tissues such as cartilage or skin.

In this study, we aimed to evaluate the different cell populations obtained from perichondrium and cartilage from both healthy individuals and microtia patients, as well as to compare progenitor cells (isolated by outgrowth) with differentiated cells (obtained by enzymatic digestion), as Koelling et al. [19] claimed that migratory cells have a progenitor character and regenerative properties. Moreover, Vinod et al. [20] showed that cells obtained by outgrowth have superior progenitor properties compared to cells obtained by fibronectin attachment. Thus, in our study, the cells obtained by outgrowth are called progenitor cells, and defining these populations may contribute to the development of bioengineered cartilage based on the use of highly proliferating progenitor cells.

The obtained results may contribute to the development of research on microtia disease by focusing on biochemical elements that differ between chondrocytes and perichondrocytes from healthy donors and those affected by the disease.

## 2. Materials and Methods

### 2.1. Cell Isolation and Cultivation

Auricular samples were obtained from the Department of Otorhinolaryngology, Head and Neck Surgery, Medical Faculty Mannheim, University of Heidelberg, Mannheim, Germany. Parents or patients gave informed written consent. This study was conducted according to the Declaration of Helsinki Principles and after permission from the Ethics Commission (2018-584-N-MA, Mannheim, Germany).

### 2.2. Isolation by Digestion

Biopsies from both healthy individuals and microtia patient donors were pre-digested with 0.5% Pronase from Streptomyces griseus (Roche, Mannheim, Germany) diluted in Dulbecco’s Modified Eagle Medium (DMEM) (Gibco, Reinach, Switzerland) at 37 °C for 1.5 h to separate perichondrium from cartilage. Healthy tissues (perichondrium and cartilage) and microtia cartilage were digested separately in collagenase solution (DMEM (Gibco, Switzerland)), 10% FBS (Gibco, Switzerland), 10 μg mL^−1^ gentamicin (Sigma-Aldrich, St. Louis, MO, USA), 0.1% collagenase from *Clostridium histolyticum* (Sigma-Aldrich, Darmstadt, Germany) overnight at 37 °C to obtain healthy chondrocytes (HCDs) and perichondrocytes (PCDs), and microtia chondrocytes (MCDs), respectively. Microtia perichondrium was treated with 1% Liberase DH (Roche, Germany) diluted in PBS for 30 min at 37 °C to obtain microtia perichondrocytes (MPDs). Then, each of the cell solutions was passed through a 100 μm cell strainer, centrifuged at 300× *g* for 10 min, resuspended in 10 mL of the medium, and seeded on 10 cm plastic dishes.

Chondrocytes obtained from healthy donors (HCDs) and microtia patients (MCDs) were cultured in a monolayer in chondrocytes expansion medium (High-Glucose DMEM (Gibco, Switzerland)), 10% FBS, 10 μg mL^−1^ gentamycin (Sigma-Aldrich, Buchs, Switzerland), 1% ITS + premix (Corning, Corning, NY, USA), 50 μg mL^−1^ L-ascorbic acid (Sigma-Aldrich, Germany), and 40 μg mL^−1^ L-proline (Sigma-Aldrich, USA). Perichondrocytes obtained from healthy donors (HPDs) and microtia patients (MPDs) were cultured in a monolayer in perichondrocytes expansion medium (DMEM and Ham’s F-12 medium (Gibco) supplemented with 10% FBS (Gibco, Switzerland) and 1% antibiotic antimycotic solution at 37 °C, 5% CO_2_ and 95% humidity.

### 2.3. Isolation by Outgrowth

Microtia chondrocytes (MCOs) and perichondrocytes (MPOs) (Table 1) were isolated by outgrowth of the tissue on T75 cell culture flasks. Cell expansion was accomplished after approximately 21 days. MCOs were cultured in chondrocytes expansion medium (High-Glucose DMEM (Gibco, Switzerland)), 10% FBS, 10 μg mL^−1^ gentamycin (Sigma-Aldrich, Buchs, Switzerland), 1% ITS + premix (Corning, USA), 50 μg mL^−1^ L-ascorbic acid (Sigma-Aldrich, Darmstadt, Germany), 40 μg mL^−1^ L-proline (Sigma-Aldrich, St. Louis, MO, USA), and MPOs were cultured in perichondrocytes medium (DMEM and Ham’s F-12 medium (Gibco, Switzerland) supplemented with 10% FBS (Gibco, Switzerland) and 1% antibiotic antimycotic solution).

### 2.4. Raman Trapping Microscopy

After reaching 80% confluency, healthy human and microtia cells were trypsinized, combined with DMEM, 10% *v*/*v* FBS, and 10 μg mL^−1^ gentamicin, and collected using centrifugation (3 min, 400× *g*). The cells were washed with PBS, fixed with 4% PFA for 15 min at 4 °C, and washed again with PBS. Then, 1 × 10^5^ fixed cells were suspended in 200 μL of PBS. Then, 25 μL were applied into a sterile μ-channel slide with a 0.17 mm thick borosilicate glass bottom (BBiospex^®^, microphotonX GmbH, Tutzing, Germany).

Raman spectroscopy was performed using the Raman microscopic-laser trapping system (Biospex^®^, microphotonX GmbH, Tutzing, Germany) with an inverted microscopic setup. Raman spectra were captured using a laser of 785 nm wavelength and 80 mW laser power (TOPTICA Photonics AG, Graefelfing, Germany) for an acquisition time of 10 sec, with 3 accumulation signals using a 60× water immersion objective (1.1 NA, 0.2 WD) (Olympus, Hamburg, Germany) with a laser spot size of 1 µm and the correction collar set to 0.17 mm. The detection of the Raman scattered photons was achieved using a diffraction grating and a Charge-Coupled Device detector (CCD, Andor, Belfast, UK). Cells were trapped in the laser focal point during the measurements using optical trapping capability. Each randomly selected cell was measured twice in two distinct locations, in the center and near the border, in order to collect both the cytoplasmic and the nuclear spectral features. Spectra were obtained from cells without damaging cell membrane integrity (Table 2).

### 2.5. Statistical Analysis of Raman Data

Processing the spectral data and statistical analysis were predefined using the mpX-RamSES software (microphotonX GmbH, Tutzing, Germany; https://www.microphotonx.com/products-1). All Raman spectra were acquired in a spectral range of 480–1800 cm^−1^, which contains most of the biological-relevant spectral bands. This was followed by a baseline correction using an asymmetric least square fit, cosmic spikes were removed, and the spectra were smoothened with a median filter. Next, the spectra were interpolated to continuous wave numbers and normalized by implementing unit vector normalization. Principal component analysis (PCA) was then applied to the datasets. PCA score plots illuminate the differences and similarities among samples, while the PCA loadings display the Raman spectral differences that are used to compare the analyzed samples [21,22]. Linear discriminant analysis (LDA), a strong tool for sample discrimination, was applied to characterize differences between samples. In LDA, linear transformations of the variables to the new discriminant functions (LDs) are computed, in which the directions of the LDs are determined in the spectral space. Moreover, mpX-RamSES was used for data visualization. Multiple comparisons of the cell groups were performed and correlated with each other. Hierarchical cluster analysis (HCA) was applied as an unsupervised statistical analysis and clustering method to separate the Raman spectra based on the different spectral observations into different clusters [23].

### 2.6. Immunofluorescence

The plastic plates (5 cm) with chondrocytes (*n* = 5 independent donors) and perichondrocytes (*n* = 5 independent donors) were washed three times with PBS for 5 min. Then, cells were fixed in acetone:methanol (1:1) at −20 °C for 5 min, and subsequently, were washed again with PBS for 5 min. Afterward, plates were blocked with 2% BSA/PBS at RT for 30 min. To visualize the expression of collagen 2, plates were incubated with an antibody: rabbit anti-collagen type 2 (Clone: ab34712, 1:100; Abcam, Switzerland) diluted in 2% BSA in PBS at 4 °C overnight. For secondary antibodies, we used donkey anti-Alexa-488 (1:400; Abcam). Subsequently, cells were co-stained with Hoechst 33,342 (1 μL/1 mL PBS; Sigma-Aldrich, Switzerland) and washed with PBS. Finally, the plates were mounted with Fluoroshield histology mounting medium (Sigma-Aldrich, Switzerland). All the pictures were taken with a Nikon Eclipse TE2000-U inverted fluorescence microscope (Nikon, Japan) equipped with Hoechst, FITC, and TRITC filters (Nikon AG, Egg, Switzerland) and processed with Photoshop 7.0 (Adobe System Inc, Munich, Germany).

### 2.7. Flow Cytometry Analysis

Chondrocytes (*n* = 3 independent donors) and perichondrocytes (*n* = 3 independent donors) were analyzed with flow cytometry. Cells (5 × 10^5^) were stained with the desired antibodies, Zombie NIR™ (1:600, BioLegend, London, UK) as live/dead dye, then rabbit anti-collagen type 2 (Clone: ab34712, 1:100; Abcam, Cambridge, UK), CD29 (Alexa-488, Clone TS2/16, 1:50, BioLegend, London, UK), and CD44 (Alexa-488, Clone BJ18, 1:50, BioLegend, London, UK). As a secondary antibody, we used donkey-anti-rabbit Alexa-488 (1:400; Abcam, Cambridge, UK). Unstained cells and cells stained with isotype-matched antibodies were used as controls. Following the incubation with the antibodies, cells were washed twice with 2 mL FACS buffer (0.5% human serum albumin, 0.5 mM EDTA in PBS) and centrifuged at 184× *g*. In the final step, the sample cells were resuspended in 0.5 mL FACS buffer, filtered into FACS tubes, and analyzed with flow cytometry on a FACS ARIA III 4 L (BD Biosciences, Wokingham, UK) provided by the Center for Microscopy and Image Analysis, University of Zurich, Zurich, Switzerland using BD FACSDiva™ software (BD Biosciences, Wokingham, UK) to control the setup, acquisition, and analysis of flow cytometry data.

The instrument setup of FACSDiva™ ensures consistent sample analysis. This process involves verification of optical detectors and fluidic stability, standardization of instrument detector settings, and establishment of color compensation settings. The flow cytometry data were analyzed using FlowJo^TM^ software (BD, Biosciences, Wokingham, UK). Forward- and side-scatter parameters were used to exclude doublets from the analysis. 

## 3. Results

**Figure 1:**
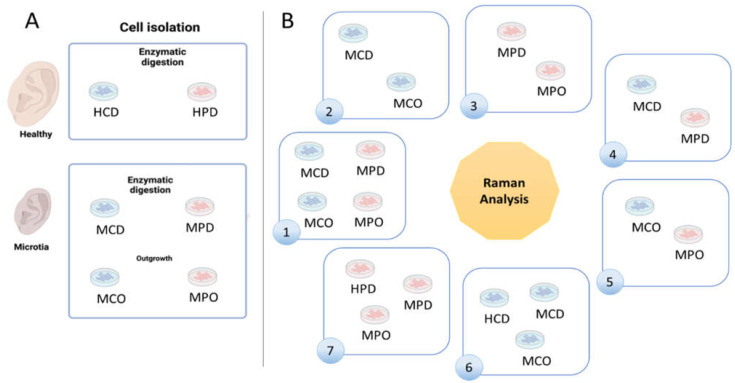
Schematic illustration of the experimental design. (**A**) Cells isolated from healthy and microtia auricular samples were enzymatically digested to obtain mature, fully differentiated cells or isolated by tissue outgrowth. (**B**) Raman spectra were collected from each individual sample. To find differences between samples, seven different comparative groups were formed and analyzed using mpX-RamSES software: healthy chondrocytes obtained by digestion (HCD), healthy perichondrocytes obtained by digestion (HPD), microtia chondrocytes obtained by digestion (MCD), microtia chondrocytes obtained by outgrowth (MCO), microtia perichondrocytes obtained by outgrowth (MPO), and microtia perichondrocytes obtained by digestion (MPD). Created with BioRender.com (accessed on 1 October 2022).

Healthy and microtia cells were isolated according to the described methods (Figure 1A). We defined 7 analysis groups (Figure 1B), which allowed for finding differences between the cell types or the different isolation methods. Group 1 shows MCD and MCO, and MPD and MPO, whereas Group 2 shows the comparison between MCD and MCO, and Group 3 the comparison between MPD and MPO. Group 4 displays the comparison between MCD and MPD, and Group 5 the comparison between MCO and MPO. HCD vs. MCD vs. MCO are compared in Group 6, and HPD vs. MPD vs. MPO in Group 7.

**Figure 2:**
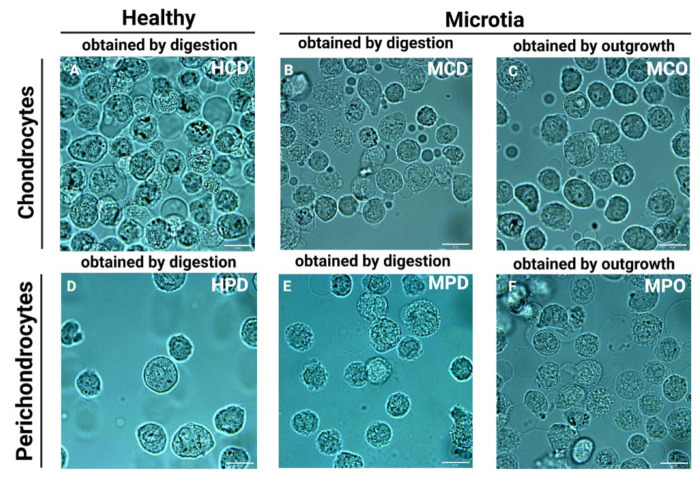
Microscopic bright field images with 60× magnification of cell suspensions in the channels used for the Raman measurements. (**A**) Healthy chondrocytes isolated by enzymatic digestion (HCD), (**B**) microtia chondrocytes isolated by enzymatic digestion (MCD), (**C**) microtia chondrocytes isolated by outgrowth (MCO), (**D**) healthy perichondrocytes obtained by enzymatic digestion (HPD), (**E**) microtia perichondrocytes isolated by enzymatic digestion (MPD), (**F**) microtia perichondrocytes isolated by outgrowth (MPO). Scale bars for macroscopic pictures: 20 µm.

Representative bright field images of the cells placed in the microchannels directly before the measurements are presented in Figure 2. For the measurement, only cells with intact cellular membranes were taken. However, the discrimination between the nucleus and cytoplasm was mostly impossible due to the small volume of the cells and the relatively high dimensions of the nuclei.

Table 3 represents spectral interpretation of Raman band assignments observed in different cell populations. All assignments of the wavenumber (cm^−1^) were analyzed with reference to Talari et al. [24] and Movasaghi et al. [25].

**Figure 3:**
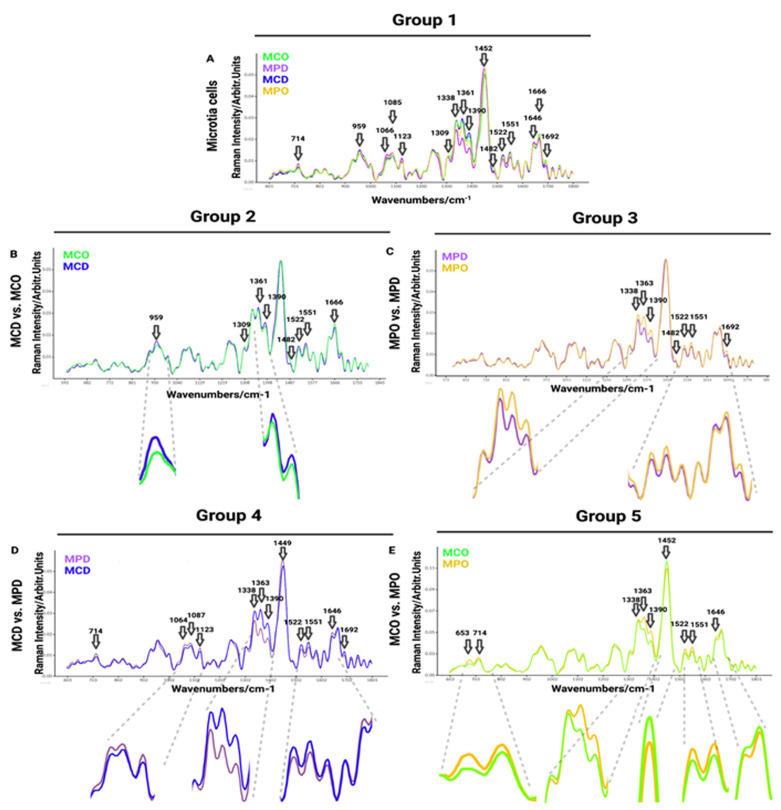
Visualization of Raman mean spectra of different cell populations: MCD (blue), MPD (purple), MCO (green), MPO (yellow). (**A**) Group 1: Comparison between MCD (*n* = 133), MCO (*n* = 177), MPD (*n* = 179), and MPO (*n* = 96), (**B**) Group 2: Comparison between MCD (*n* = 133) and MCO (*n* = 177), (**C**) Group 3: Comparison of MPD (*n* = 179) vs. MPO (*n* = 96), (**D**) Group 4: Comparison between MCD (*n* = 133) and MPD (*n* = 176), (**E**) Group 5: Comparison between MCO (*n* = 375) and MPO (*n* = 220). Black arrows indicate selected Raman peaks with the strongest visible difference between the compared groups.

**Figure 4:**
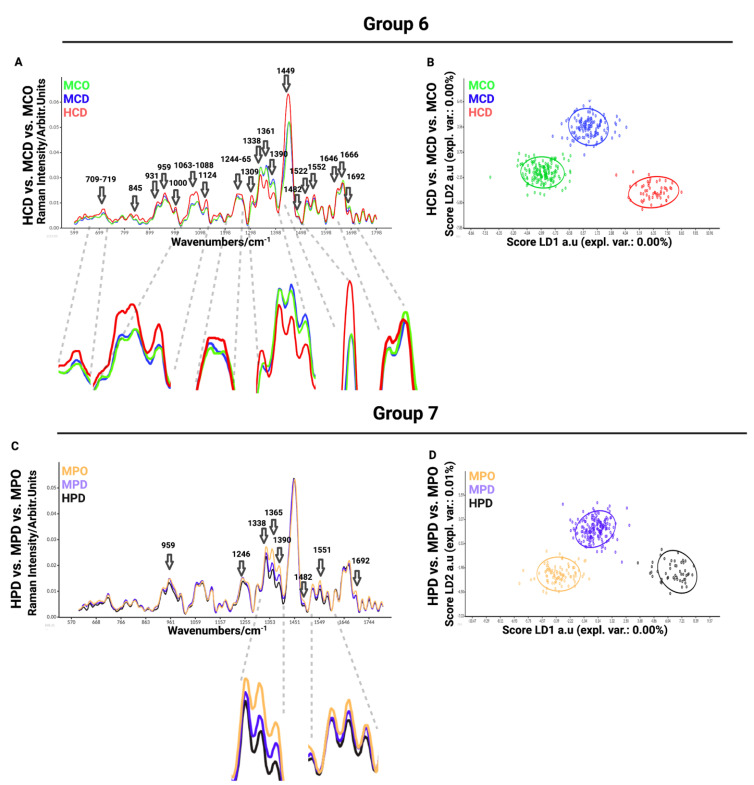
Visualization of Raman mean spectra (**A**) Group 6: Raman means spectra for healthy chondrocytes (HCD) (red), (MCD) (blue), and (MCO) (green), (**B**) LDA corresponding to measurements of the microtia chondrocytes MCD (blue) (*n* = 133), healthy chondrocytes HCD (red) (*n* = 174), and microtia chondrocytes obtained by outgrowth MCO (green) (*n* = 177), (**C**) Group 7: Raman mean spectra for healthy perichondrocytes (HPD) (black), microtia perichondrocytes (purple), microtia perichondrocytes obtained by outgrowth (yellow), (**D**) LDA corresponding to measurements of the microtia perichondrocytes MPD (purple) (*n* = 179), healthy perichondrocytes HPD (black) (*n* = 140), and microtia perichondrocytes obtained by outgrowth MPO (yellow) (*n* = 96).

Raman spectra comparisons between distinct microtia cells are presented in Figure 3, whereas microtia and healthy cell spectra are displayed in Figure 4. In addition, Raman mean spectra with standard deviation are included in Figure S1

The first comparison of Raman spectra was performed between independent donors of MPO, MPD, MCD, and MCO (Figure 3A). This was done to visualize and find the most prominent peaks in the spectra, which were then compared in detail in smaller groups. The MCD showed increased Raman bands with respect to other groups at the following spectra: 959 cm^−1^ (symmetric stretching vibration of v_1_PO_4_^3−^ (phosphate of HA)/hydroxyapatite)), 1361 cm^−1^ (guanine (N_7_, B, Z-marker)), 1375–1410 cm^−1^ (GAGs), 1522 cm^−1^ (-C=C- carotenoids), 1552 cm^−1^ (tryptophan). The MPD mean spectrum shows higher peaks at 713 cm^−1^ (AA methionine), 1066 cm^−1^ (proline: collagen assignment), 1123 cm^−1^ (C-N proteins), and 1646 cm^−1^ (amide I bond) in comparison to other groups. The MCO exhibits increased Raman bands over other groups at 1482 cm^−1^ (amide III), 1666 cm^−1^ (collagen), and 1692 cm^−1^ (in-plane double-end vibrations of bases) spectra (Figure 3A).

In the second group (Figure 3B), we compared chondrocytes obtained by digestion (MCD) and chondrocytes obtained by outgrowth (MCO) to see if the isolation methods of microtia chondrocytes affect the Raman spectra, thus, to reveal if the population defined as progenitor isolated by outgrowth express different biomolecules than the whole chondrocytes population obtained by digestion. The collected results exhibit minor differences in Raman spectra (Figure 3B). The strongest spectral differences are observed at 959 cm^−1^ (symmetric stretching vibration of v_1_PO_4_^3−^ (phosphate of HA)/hydroxyapatite), ~1313 cm^−1^ (CH_3_/CH_2_ twisting or bending mode of lipid/collagen), 1389 cm^−1^ (CH bending), 1481 cm^−1^ (amide III), 1521 cm^−1^ (-C=C- carotenoids), 1552 cm^−1^ (tryptophan), 1666 cm^−1^ (collagen), and 1692 cm^−1^ (in-plane double-end vibrations of bases) (Figure 3B).

The third comparison was performed between perichondrocytes obtained by outgrowth (MPO) and perichondrocytes obtained by digestion (MPD) (Figure 3C), aiming to identify the differences between the isolation methods. Collected results showed a few small differences in Raman spectra between the cell populations (Figure 3C) at 1339 cm^−1^ (tryptophan), 1363 cm^−1^ (guanine (N_7_, B, Z-marker)), 1375–1410 cm^−1^ (GAGs), 1484 cm^−1^ (amide III), 1522 cm^−1^ -C=C- carotenoids), 1552 cm^−1^ (tryptophan), 1692 cm^−1^ (in-plane double-end vibrations of bases).

The fourth cell grouping between microtia chondrocytes obtained by digestion (MCD) and perichondrocytes obtained by digestion (MPD) was designed for the distinction between the chondrocytes and perichondrocytes. The analysis showed differences in Raman spectra at 714 cm^−1^ (AA methionine), 1063 cm^−1^ (SO_3_^−^ stretching, GAGs (chondroitin sulfate)), 1087 cm^−1^ (C-C stretch), 1123 cm^−1^ (C-N) proteins (protein assignment), 1338 cm^−1^ (collagen (CH_3_CH_2_ waging mode)), 1363 cm^−1^ (guanine (N_7_, B, Z-marker)), 1375–1410 cm−1 (GAGs), 1449 cm^−1^ (C-H vibrations), 1521 cm^−1^ (-C=C- carotenoids), 1551 cm^−1^ (tryptophan), 1646 cm^−1^ (amide I bond (protein bonds)), 1692 cm^−1^ (in-plane double-end vibrations of bases) (Figure 3D).

The comparison of healthy chondrocytes (HCD) (*n* = 174; *n* = 6 donors) with healthy perichondrocytes (HPD) (*n* = 140; *n* = 5 donors) (Figure S5) revealed differences in the region of 784 cm^−1^ (nucleotide conformation), 821 cm^−1^ (proline), 1338 cm^−1^ (collagen (CH3CH2 waging mode)), 1361 cm^−1^ (tryptophan), 1390 cm^−1^ (CH bending), 1449 cm^−1^ (C-H vibrations), 1522, cm^−1^ (-C=C- carotenoids), 1552 cm^−1^ (tryptophan), 1613 cm^−1^ (amide 1 band of proteins), 1646 cm^−1^ (amide 1 (α helix)), and 1666 cm^−1^ (collagen). The most visible differences were recorded at 1613 cm^−1^ (amide 1 band of proteins), 1646 cm^−1^ (amide 1 (α helix)), and 1666 cm^−1^ (collagen) wavelength. The differences between HCD and HPC (Figure S5) are not the same as for MCD and MPD or MCO and MPO (Figure 3D). In particular, in the regions between 1338 cm^−1^ and 1390 cm^−1^, MCD shows higher contributions than MPD. This is not visible in the corresponding healthy HCD and HPD, which show the same spectral peaks (Figure S5). In contrast, in the region between 1613 cm^−1^ and 1666 cm^−1^, MCD and MPD do not differ significantly, whereas HCD and HPC display a considerable difference with a higher contribution of the perichondrocytes (Figure S5).

In the fifth comparison between microtia chondrocytes obtained by outgrowth culture (MCO) and perichondrocytes obtained by outgrowth culture (MPO), we aimed to show the differences between the progenitor cells obtained by outgrowth from the cartilage part and progenitor cells localized in the perichondrium (Figure 3E). In the fifth analysis, we observed differences in Raman spectra at 653/714 cm^−1^ (AA methionine), 1338 cm^−1^ (collagen (CH_3_CH_2_ waging mode)), 1362 cm^−1^ (guanine (N_7_, B, Z-marker)), 1375–1410 cm−1 (GAGs), 1452 cm^−1^ (guanine, Adenine (DNA, RNA)), 1522 cm^−1^ -C=C- carotenoids), 1552 cm^−1^ (tryptophan), and 1646 cm^−1^ (amide I bond) (Figure 3E).

In the sixth comparison between healthy chondrocytes (HCD) and microtia chondrocytes obtained by digestion (MCD) or outgrowth culture (MCO), we aimed to define the differences between healthy and microtia cells (Figure 4A). This analysis is important for defining the differences and furthering the cause of the disease itself. We observed differences in Raman spectra at 709–716 cm^−1^ (AA methionine), 717–719 cm^−1^ (phospholipids, lipids, adenine), 845 cm^−1^ (polysaccharide structure), 931 cm^−1^ (carbohydrates peak for solutions and solids), 959 cm^−1^ (symmetric stretching vibration of v_1_PO_4_^3−^ (phosphate of HA)/hydroxyapatite), 1000–1004 cm^−1^ (phenylalanine of collagen + collagen assignment), 1060 cm^−1^ (C-C skeletal stretching), 1066 cm^−1^ (proline (collagen assignment)), 1088 cm^−1^ (C-C stretch), 1124 cm^−1^ (v(C-C) skeletal of acyl backbone in lipids), 1244–1255 cm^−1^ (lipids), 1264 cm^−1^ (triglycerides (fatty acids)), 1265 cm^−1^ (amide III of collagen), 1338 cm^−1^ (collagen (CH_3_CH_2_ waging mode)), 1360 cm^−1^ (tryptophan), 1389 cm^−1^ (CH bending), 1449 cm^−1^ (C-H vibrations), 1482 cm^−1^ (Amide II), 1521 cm^−1^ (-C=C- carotenoids), 1552 cm^−1^ (tryptophan), 1645 cm^−1^ (amide I (α helix)), 1666 cm^−1^ (collagen), and 1692 cm^−1^ (in-plane double-end vibrations of bases). The most prominent differences observed in the spectra were detected at 1063–1088 cm^−1^, corresponding to collagen, GAGs, and lipids (Figure 4A). LDA maximized the data separation between the groups and successfully discriminated between healthy and microtia samples (Figure 4B).

The last, seventh comparison was between healthy perichondrocytes (HPD), microtia perichondrocytes obtained by digestion (MPD), and microtia perichondrocytes obtained by outgrowth culture (MPO) (Figure 4C). This analysis was designed to define the differences between healthy and microtia perichondrocytes, thus allowing us to better understand the disease pattern. The comparison showed differences at 959 cm^−1^ (symmetric stretching vibration of v_1_PO_4_^3−^(phosphate of HA)/hydroxyapatite), 1246 cm^−1^ (amide III (of collagen)), 1338 cm^−1^ (collagen (CH_3_CH_2_ waging mode)), 1365 cm^−1^ (tryptophan), 1375–1410 cm^−1^ (GAGs), 1482 cm^−1^ (amide III), 1522 cm^−1^ (-C=C- carotenoids), 1552 cm^−1^ (tryptophan), 1645 cm^−1^ (amide I (α helix)), 1666 cm^−1^ (collagen), and 1692 cm^−1^ (in-plane double-end vibrations of bases) (Figure 4D). The most visible differences were recorded at 1338 cm^−1^ (collagen), 1365 cm^−1^ (tryptophan), and 1375–1410 cm^−1^ (GAGs) wavelengths.

To classify the similarities and variations between the examined data, we performed principal component analysis (PCA) (Figure S4), which is a multivariate analysis method that can group related individuals based on their PCA scores and can classify the interrelations among the examined groups. However, in our experiment, PCA scores were not separating measured groups. Therefore, advanced linear discriminant analysis (LDA) revealed the differences between samples by the supervised machine learning algorithm created to maximize the separation of the groups based on the group variance (Figure 4D). This is an additional tool to visualize better group separation. By inputting successive readings and measurements into the system, the Raman system is able to learn to separate groups and, thus, automatically assign the measured cells to a particular group based on Raman spectra in the future.

In Figure 3 and Figure 4, the data are presented as smoothed spectra. Smoothing did not affect the interpretation (see the raw, non-smoothed spectra in Figure S2).

Raman principle component analysis (PCA) was used with the aim of visualizing the difference between the studied cell populations. The score plots show the PC1 to PC2 correlation of the analysis groups shown in Figure 3 and Figure 4. We observed that the data point clouds were almost completely overlapping, indicating a high global similarity of the compared cell populations (Figure S3A–G). The specific differences at the defined wavelength discussed before resulted in “merged” or “diluted” in the huge number of spectral regions with no difference between the populations. Therefore, PCA was demonstrated not to be suitable for highlighting the singularity of the cell groups (Figure S3).

For this reason, we analyzed the loading plots of each principal component (Figure S4) by highlighting with arrows the same assignments as in Figure 3 and Figure 4. In particular, PC1 and PC2 show the highest and relatively similar contribution to the variations observed between the different groups.

We next sought to analyze the variation in the spectra within specific cell populations used in our study. We hypothesized that the variation could be due to the presence of different subpopulations. For this, we implemented the hierarchical cluster analysis (HCA) on the example of a microtia MCD sample with merged donors (*n* = 4) (Figure S6A) and separated the population into 3 clusters (Figure S6B). The mean spectra of the obtained subpopulations (Figure S6C) show high variability in particular spectral regions (asterisks) within the MCD cell population, which results in distinct data point clouds in the PCA (Figure S6D). The same variation was observed within the single donors (Figure S7), meaning that microtia chondrocytes are a highly heterogeneous cell population.

Further, we assessed whether the MCD clusters shared molecular characteristics with the healthy counterparts, HCD. We performed a combined analysis of the MCD clusters (*n* = 4) shown in Figure S6 with the merged HCD (black line) donors (*n* = 6) (Figure S8). We found that two MCD subpopulations (cluster 1, green, and cluster 3, red) were more similar to the healthy HCD cells and one subpopulation (cluster 2, blue), which differed more markedly from the healthy cells. This finding implicates that the microtia group contains cell subpopulations with high similarity to the healthy cells.

**Figure 5:**
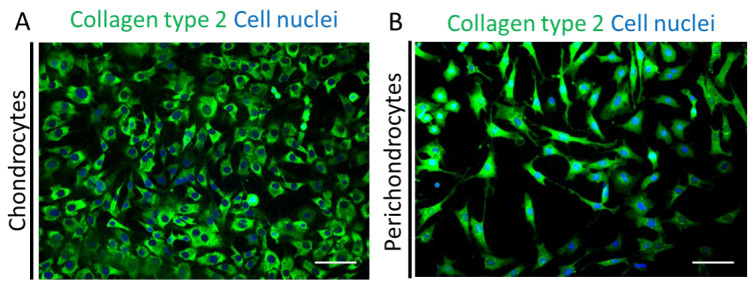
Immunofluorescence stainings of collagen type 2 in human healthy chondrocytes and perichondrocytes. Human collagen type 2 (green) co-stained with Hoechst (blue) to visualize the collagen presence in the healthy chondrocytes (**A**) and perichondrocytes (**B**) on tissue culture plastic; representative for *n* = 5 donors. Scale bar: 100 μm.

Next, we performed immunofluorescence stainings for human collagen type 2 (green) co-stained with Hoechst (blue) in the healthy chondrocytes (Figure 5A) and perichondrocytes (Figure 5B) on tissue culture plastic. The analysis showed a different morphology of both cell types and confirmed a high expression of collagen type 2 extracellular matrix protein in chondrocytes and perichondrocytes.

**Figure 6:**
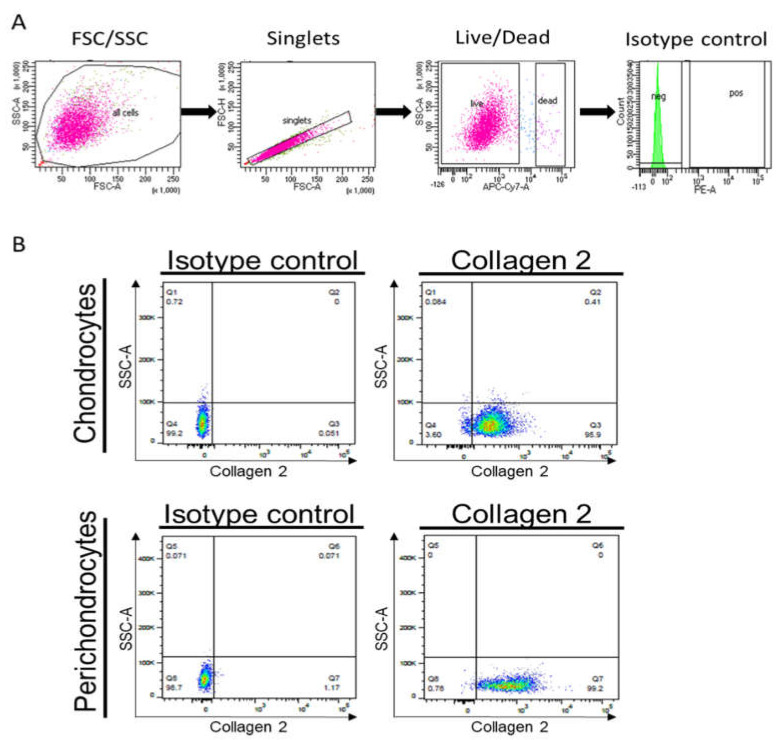
Flow cytometric analysis for the expression of collagen 2 in healthy chondrocytes and perichondrocytes. (**A**) Gating strategy for flow cytometry including FSC/SSC (first graph, pink), singlets (second graph, pink), live/dead (third graph, pink), and isotype control (fourth graph, green) (**B**) respective isotype controls or collagen type 2 expression in cultured chondrocytes and perichondrocytes.

Moreover, we performed a flow cytometric analysis to assess collagen 2 protein levels in cultured cells. Whereas Figure 6A demonstrates the gating strategy, Figure 6B confirms the expression of collagen 2 in healthy chondrocytes and perichondrocytes.

Further, we analyzed the expression of chondrocyte markers, including CD44 and CD29. The hyaluronan receptor CD44 serves as the critical link for the retention of hyaluronan–proteoglycan aggregates to the chondrocyte cell surface, whereas CD29 acts as a marker of chondrogenic potential. We observed similar levels of CD44 and CD29 in healthy chondrocytes (HCD) and healthy (HPD)/microtia perichondrocytes isolated by the digestion (MPD) or outgrowth method (MPO) (Figure S9). However, the expression of CD29 was reduced in microtia chondrocytes isolated with either method (MCD and MCO).

## 4. Discussion

The characterization of cells obtained from microtia patients is challenging because of the limited number of cells obtained from small biopsies and the high similarity of cells harvested from cartilage and perichondrium. This makes the identification of specific cell populations by applying standard techniques such as flow cytometry analysis rather impossible.

Recently, Raman spectroscopy appeared as a novel, non-invasive, and label-free diagnostic tool to detect distinctive biochemical features of single cells and/or tissues [24,25,26]. Understanding the biochemical fingerprints of healthy and microtia cells, chondrocytes, and perichondrocytes, and between progenitor cells and differentiated cells, would allow discriminating between them and performing specific functionality assays [27,28]. One example of using Raman spectroscopy in disease diagnosis is proposed by Kumar et al., who characterized osteoarthritic chondrocytes [29]. They showed that the decrease in protein and nucleic acid content is a clear diagnostic indicator of high-grade osteoarthritis [29,30]. Recently, Raman spectra of cartilage [31,32,33] and bone [34] have been studied; however, little is known about the biochemical characteristics of these tissues at the cellular level. No studies so far have compared healthy and microtia cartilage and perichondrium-derived cells.

In the present study, the analysis of Raman mean spectra showed that microtia cells comprise a heterogeneous cell population, probably representing different stages of disease. The HCA analysis showed a relevant heterogeneity, in particular, in spectral regions between both the single cells of the same donor and the different donors. PCA analysis of the resulting clusters displayed, in both cases, distinguishable subpopulations. This denotes that the origin of variability resides primarily in the cell population of every single donor and/or in the precision of laser targeting, which cannot distinguish between the cytoplasma, nucleus, and other organelles. However, donor-to-donor variance cannot be discounted and may be linked to age, gender, and disease sources [35,36]. This is a well-known challenge in cells and tissues originating from human sources [35,36]. Therefore, we included in our study as many donors as possible for the analysis of differences and similarities in the biochemical composition of cells isolated with different methods.

For example, the analysis of microtia chondrocytes obtained by enzymatic digestion with microtia chondrocytes obtained by outgrowth apparently showed no significant differences in the Raman spectral bands, suggesting a high degree of similarity between these two cell populations. However, the most prominent difference in the band intensity was measured at 959 cm^−1^, which correlates with phosphate ion interactions but also marks the presence of the hydroxyapatite biomolecule. In this respect, the microtia chondrocytes obtained by digestion exhibited a higher hydroxyapatite peak than the cells obtained by outgrowth. The relevance of this observation is illustrated by the study by Zucchelli et al., which showed that microtic ear cartilage contained small blood capillaries in the chondrium [28] which were not present in normal, healthy ear cartilage tissue. The presence of capillaries may cause hypertrophy, which, in turn, leads to cartilage mineralization [27] and hydroxyapatite deposition. Microtia chondrocytes obtained by outgrowth have progenitor characteristics and, thus, are in the early stage of differentiation. Cells obtained by digestion display the whole population of chondrocyte cells, which also contain dedifferentiated chondrocytes in the very late stage before they undergo apoptosis. In addition, cells seeded on plastic plates can differentiate during culture time, so another aspect affecting the level of hydroxyapatite in the cells would be the time of the culture on the dish. All these factors can possibly affect the hydroxyapatite level.

Another difference between the cell populations was recorded in the collagen assignments. Here, we faced one of the limitations of Raman spectroscopy, which enables studying cellular biomolecules as a whole but is not the best technique for protein-targeted analysis. In fact, the assignment of different biomolecules can overlap in the same mean spectra. However, since collagen is reported as the most abundant protein in cartilage [35], it can be assumed that in the peaks indicating collagen, the contribution of this molecule is more important than the contribution of any other protein. In order to confirm the high expression of collagen, in particular collagen type 2, we visualized it in the cells using immunofluorescence staining and flow cytometry, proving the in-, and especially, the extra-cellular presence of collagen in healthy chondrocytes and perichondrocytes. The bulk of collagen being located outside the cell in the extracellular matrix (ECM) was previously reported [37].

Taking this into account, we observed that microtia and healthy chondrocytes (obtained by enzymatic digestion or by outgrowth) show higher spectral contributions of collagen compared to perichondrocytes. If cells from the perichondrium have a lower collagen contribution than cells located in the cartilage, they express less ECM.

Lower deposition of ECM in the perichondrium than in the cartilage leads to different mechanical properties. In fact, the study reported by Sun et al., describing the mechanical properties of rabbit auricular cartilage with and without perichondrium using tensile and compressive tests, reveals that perichondrium contributes to the mechanical properties of the whole ear [38]. The presence of perichondrium increases the ability to bear compressive forces and enhances ear elasticity [36]. Identifying the differences between perichondrial cells and chondrocytes in the future could help during the isolation of these cells and subsequent selection for engineering ear tissues. Finally, comparing the same cell types but isolated by outgrowing (with progenitor character), we recorded a similarity in the biochemical characteristics of measured cells. This may indicate that progenitor cells from both perichondrium and cartilage may have a common origin, resulting in similar properties and functions. In addition, we suggest collagen to be a useful biomarker for distinguishing between populations obtained from the cartilage and perichondrium in healthy and microtia patients. Importantly, two recent reviews confirmed the close relationship between the ECM and regeneration [39,40].

Unfortunately, there are only a few studies that have compared microtia chondrocytes to healthy human chondrocytes. For example, it was reported that microtia cartilage has a more disorganized microscopic appearance; however, gene expression profiles and biochemical composition are similar to normal auricular cartilage [41]. Otto et al. demonstrated, in contrast, that microtia cells express higher levels of ECM proteins, especially collagen type II, than healthy cells [42]. These findings were also confirmed by our Raman analysis.

Intriguingly, microtia chondrocytes exhibit significantly lower levels of SO_3_^−^ stretching and GAGs (chondroitin sulfate) than healthy chondrocytes. GAGs are very important molecules responsible for building the cartilage extracellular matrix and crucial to bearing the compressive forces in healthy cartilage. We can assume that patients suffering from microtia have a lower matrix deposition and, thus, less resistance to compression. Moreover, GAG disturbance can affect cell adhesion, proliferation, or migration [43], which can cause abnormal cartilage development.

Further, between microtia perichondrocytes isolated by enzymatic digestion and by outgrowth, we recorded the only visible differences in a small spectral region assigned to collagen, guanine, and GAGs. All the other peaks are quite similar, suggesting that both cell populations have a high biochemical similarity. Nevertheless, the different deposition of collagen could indicate that cells obtained by outgrowth, classified as progenitors, have a capacity for an extensive production of the matrix.

Interestingly, chondrocytes derived from microtia patients exhibited lower lipid concentrations in comparison to healthy chondrocytes. It seems that the capacity of chondrocytes to store and synthesize fats and lipids is an important factor in cell metabolism [44]. It was reported that alterations in lipid metabolism in chondrocytes and fat deposition in the human joint correlate directly with disease. One example of abnormal fat storage in the chondrocytes occurs in the early stages of osteoarthritis [30,45]. Thus, the differences in lipid content can be used to discriminate the healthy chondrocytes from microtia-derived chondrocytes. Moreover, further examination of the metabolic processes of the microtia cells may lead to finding the cause of the abnormal ear development and, thus, therapies based on the alteration of the non-functioning processes.

It would be highly interesting in the future to sort the different cell clusters observed by Raman spectroscopy in microtia samples and to characterize them in more detail. We believe that the identification of the relationship between molecular phenotypes and the function of microtia cells will facilitate uncovering the mechanism of microtia and the study of potential therapeutic targets.

## 5. Conclusions

Micro-Raman spectroscopy can give us a unique opportunity to understand the phenotypic nature of the analyzed cells. Our findings indicate the Raman spectra changes between the microtia and healthy cells as well as between human chondrocytes and human perichondrocytes. The high sensitivity of the presented technique allows for the detection of various patterns of molecules present in the analyzed cells. Due to the unique molecular characteristics of collagen and fatty acid contents, we could clearly discriminate between microtia chondrocytes and healthy cells. Furthermore, we suggest that collagen is a useful biomarker to distinguish between cell populations obtained from the cartilage and perichondrium.

## Figures and Tables

**Table 1:** Table with acronyms and full names.

Abbreviation.	Name
HCD	Healthy Chondrocytes obtained by enzymatic Digestion
MCD	Microtia Chondrocytes obtained by enzymatic Digestion
HPD	Healthy Perichondrocytes obtained by enzymatic Digestion
MPD	Microtia Perichondrocytes obtained by enzymatic Digestion
MCO	Microtia Chondrocytes obtained by Outgrowth
MPO	Microtia Perichondrocytes obtained by Outgrowth

**Table 2:** Number of donors and measured spots in the compared groups during Raman spectroscopy analysis.

Group:	Abbreviation:	Donors:	Number of Measured Spots in the Cells (in Total):
1	MCO	6	177
	MCD	4	133
	MPO	3	96
	MPD	5	179
2	MCD	4	133
	MCO	6	177
3	MPO	3	96
	MPD	5	179
4	MCD	4	133
	MPD	5	179
5	MCO	8	375
	MPO	6	220
6	MCD	4	133
	MCO	6	177
	HCD	6	174
7	MPO	3	96
	MPD	5	179
	HPD	5	140

**Table 3:** Spectral interpretation of the assignment of Raman spectra observed between cell populations. (‘-‘: lack of differences; ‘1′: small difference; ‘2′: strong difference; ‘3′: very strong difference). The plus symbol and color next to the numbers indicate the surplus of the group that is in first place in the table. The minus symbol and color indicate the surplus of the second group listed in the table. Assignments of the wavenumber (cm^−1^) were prepared in reference to Talari et al. [24] and Movasaghi et al. [25].

Wavenumber (cm^−1^)	Assignment	Ref.	Gr.2 MCO vs. MCD	Gr.3 MPD vs. MPO	Gr.4 MPD vs. MCD	Gr.5 MCO vs. MPO	Gr.6 HCD vs. MCO	Gr.6 HCD vs. MCD	Gr.7 HPD vs. MPO	Gr.7 HPD vs. MPD
~653/714	AA methionine	[25]	1	-	1	1	1	1	-	-
~717	Phospholipids, lipids, adenine	[24]	-	-	-	-	1	1	-	-
~845	Polysaccharide structure	[25]	-	-	-	-	1	1	-	-
~931	Carbohydrates peak for solutions and solids	[25]	1	-	-	-	1	1	-	-
~959	Symmetric stretching vibration of ν1PO_4_^3−^ (phosphate of HA)/hydroxyapatite	[25]	2	-	-	-	2	1	1	1
~1063	SO_3_^−^ stretching; GAGs (chondroitin sulfate)	[25]	-	-	1	-	3	3	-	-
~1066	Proline (collagen assignment)	[25]	-	-	1	-	3	3	-	-
~1083	C-N stretching made of proteins and lipid	[25]	-	-	1	-	3	3	-	-
~1124	*v*(C-C) skeletal of acly backbone in lipids	[25]	-	-	1	-	2	2	1	1
~1246	Amide III (of collagen)	[25]	-	-	-	-	1	1	-	-
~1264	Triglycerides (fatty acids)	[25]	-	-	-	-	2	2	-	-
~1338	Collagen (CH_3_CH_2_ waging mode)	[25]	-	2	3	1	3	3	3	1
~1362	Guanine (n7, B, Z-marker)	[25]	1	2	3	3	3	3	3	2
~1375–1410	GAGs	[26]	-	1	3	3	3	3	3	1
~1452	CH2CH3 deformation (collagen assignment)	[24]	-	-	1	2	3	3	-	-
~1482	Amide II	[24]	1	1			1	-	1	-
~1522	-C=C- carotenoids	[24]	1	1	1	1	2	2	2	1
~1552	Tryptophan	[25]	1	1	1	1	2	1	1	-
~1646	Amide I bond (protein bonds)	[25]	-	-	1	1	1	1	1	-
~1666	Collagen	[25]	1	-	-	-	1	1	1	-
~1692	In-plane double-end vibrations of bases	[25]	-	1	1	-	1	1	1	-

## Data Availability

All data are available in the main text or Supplementary Materials.

## Supplementary Materials

The following supporting information can be downloaded at https://www.mdpi.com/article/10.3390/biomedicines11092588/s1, Figure S1: Visualization of Raman mean spectra with a standard deviation of different cell populations; Figure S2: Visualization of HCD vs MCD Raman mean spectra without and with smoothing; Figure S3: Visualization of Raman principle component analysis (PCA) in different cell populations; Figure S4: Visualization of Raman component (PC1/PC2/PC3) loadings plots of different cell populations; Figure S5: Visualization of Raman mean spectra for healthy chondrocytes (HCD) (red) (*n* = 174; *n* = 6 donors) with healthy perichondrocytes HPD (black) (*n* = 140; *n* = 5 donors); Figure S6: HCA and PCA of microtia chondrocytes; Figure S7: HCA and PCA of microtia chondrocytes; Figure S8: Comparison of putative MCD subpopulation with healthy chondrocytes; Figure S9: Flow cytometric analysis of the expression of CD44 and CD29 markers in healthy and microtia chondrocytes and perichondrocytes isolated by digestion or outgrowth.
