# Supplementary material for: Characterization of Distinct Chondrogenic Cell Populations of Patients Suffering from Microtia Using Single-Cell Micro-Raman Spectroscopy
Source: Biomedicines. 2023 Sep 21;11(9):2588. doi: 10.3390/biomedicines11092588 (PMC10526501; doi:10.3390/biomedicines11092588)
Supplement: Supplementary file 1 [file biomedicines-11-02588-s001.zip › biomedicines-2518201-supplementary.pdf]

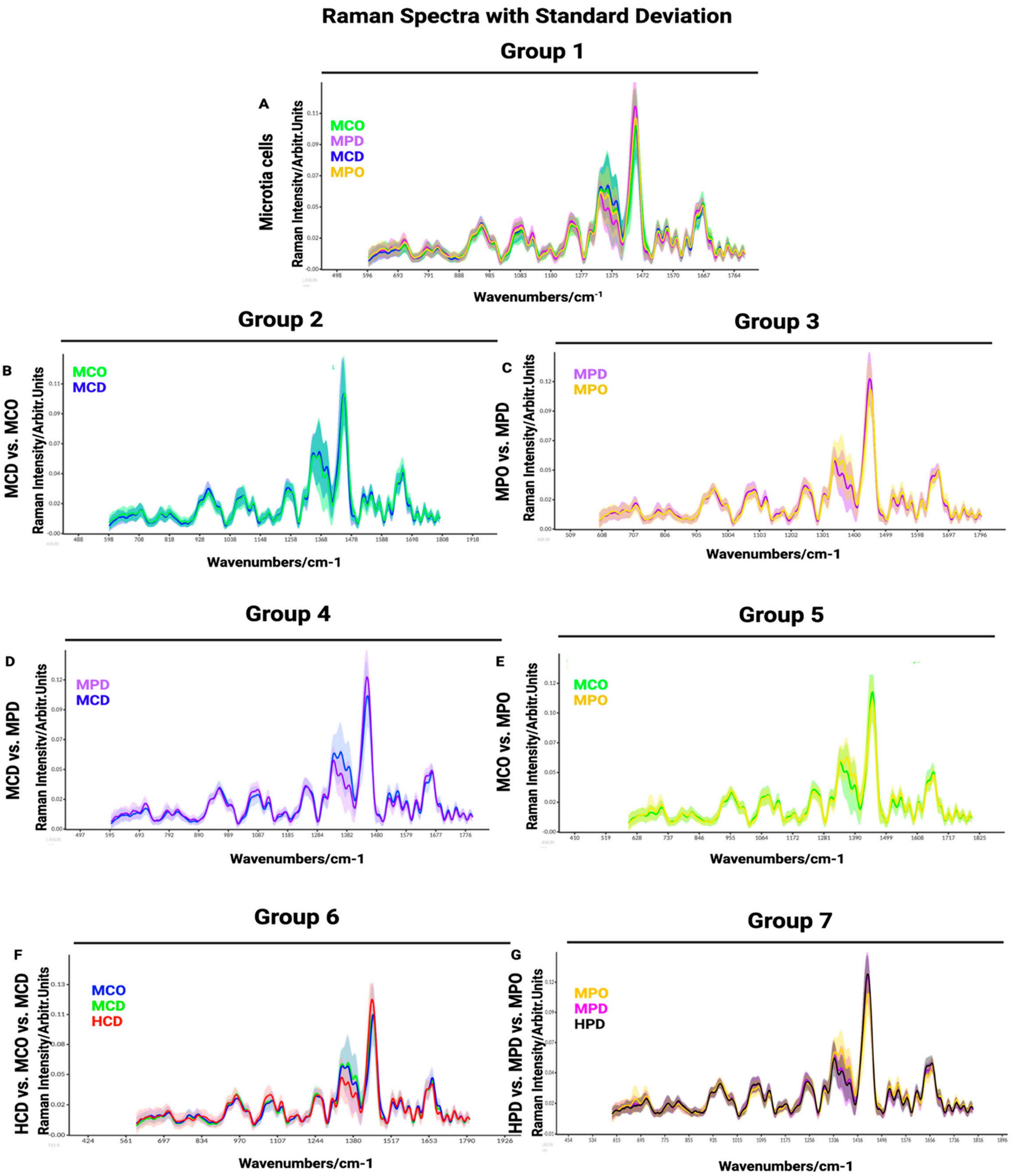

Figure S1. Visualization of Raman mean spectra with a standard deviation of different cell populations. (**A**) Group 1: Comparison between MCD (n=133), MCO (n=177), MPD (n=179) and MPO (n=96), (**B**) Group 2: Comparison between MCD (n=133) vs. MCO (n=177), (**C**) Group 3: Comparison of MPD (n=179) vs. MPO (n=96), (**D**) Group 4: Comparison between MCD (n=133) vs. MPD (n=176), (**E**) Group 5: Comparison between MCO (n=375) vs. MPO (n=220). (**F**) Group 6: Comparison between HCD (n=174) vs. MCD (n=133) vs. MCO (n=177), (**G**) Group 7: Comparison between HPD (n=140) vs. MPD (n=179) vs. MPO (n=96).

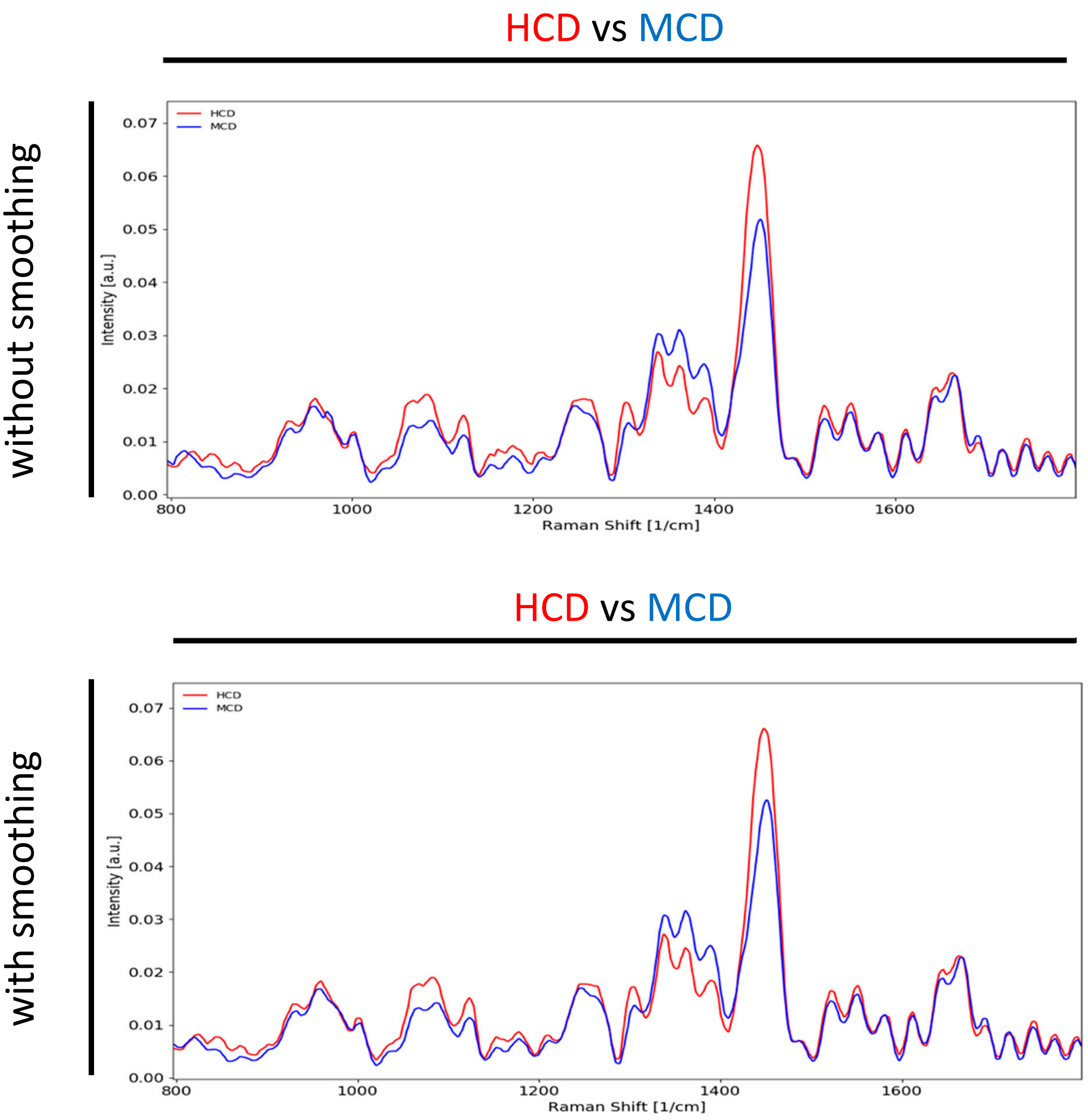

Figure S2. **Visualization of HCD vs MCD Raman mean spectra without and with smoothing.**

PCA: PC1 x PC2

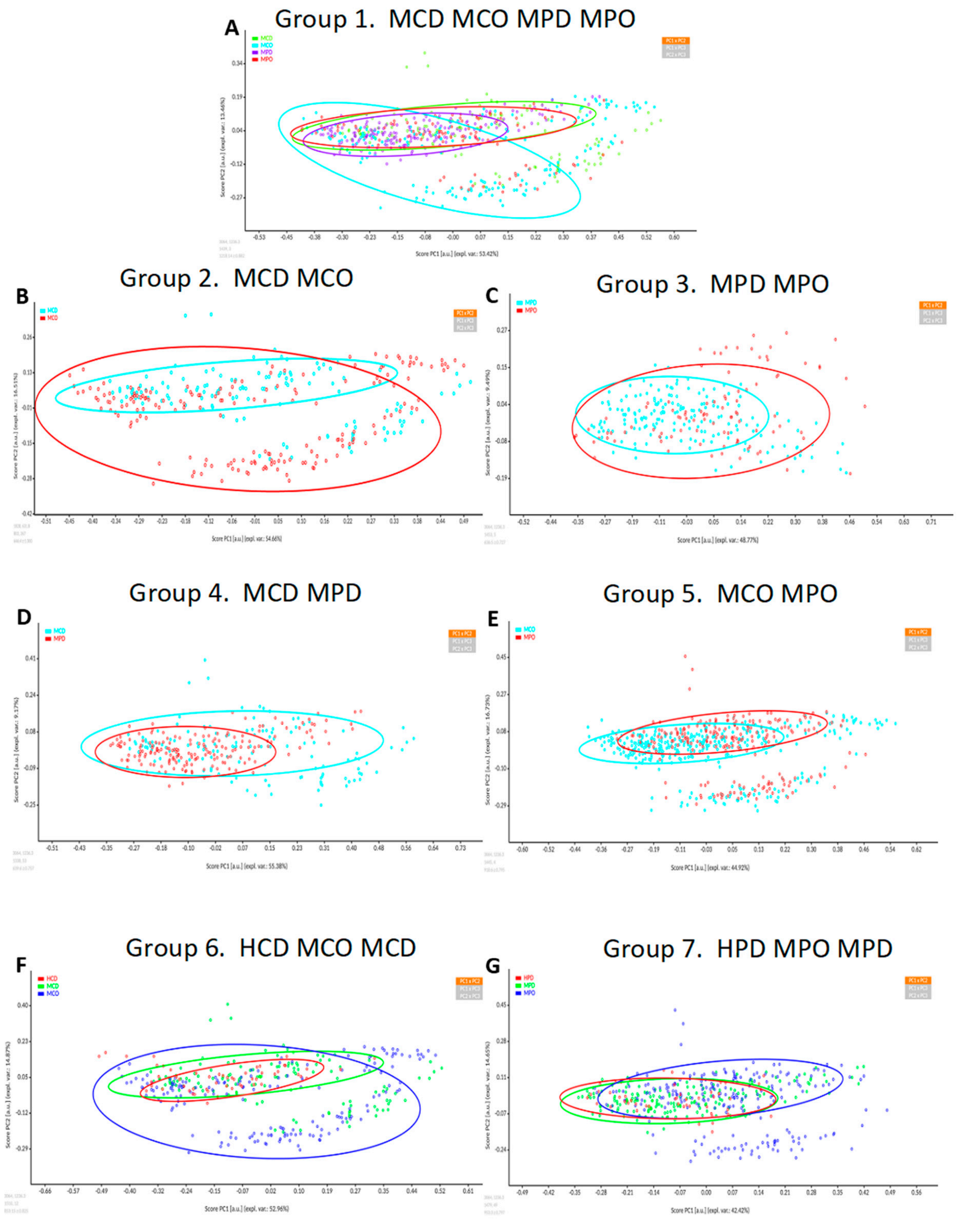

Figure S3. Visualization of Raman principle component analysis (PCA) in different cell populations. Results shows the PC1 to PC2 correlation. (**A**) Group 1: Comparison between MCD (n=133), MCO (n=177), MPD (n=179) and MPO (n=96), (**B**) Group 2: Comparison between MCD (n=133) vs. MCO (n=177), (**C**) Group 3: Comparison of MPD (n=179) vs. MPO (n=96), (**D**) Group 4: Comparison between MCD (n=133) vs. MPD (n=176), (**E**) Group 5: Comparison between MCO (n=375) vs. MPO (n=220), (**F**) Group 6: Comparison between HCD (n=174) vs. MCD (n=133) vs. MCO (n=177), (**G**) Group 7: Comparison between HPD (n=140) vs. MPD (n=179) vs. MPO (n=96).

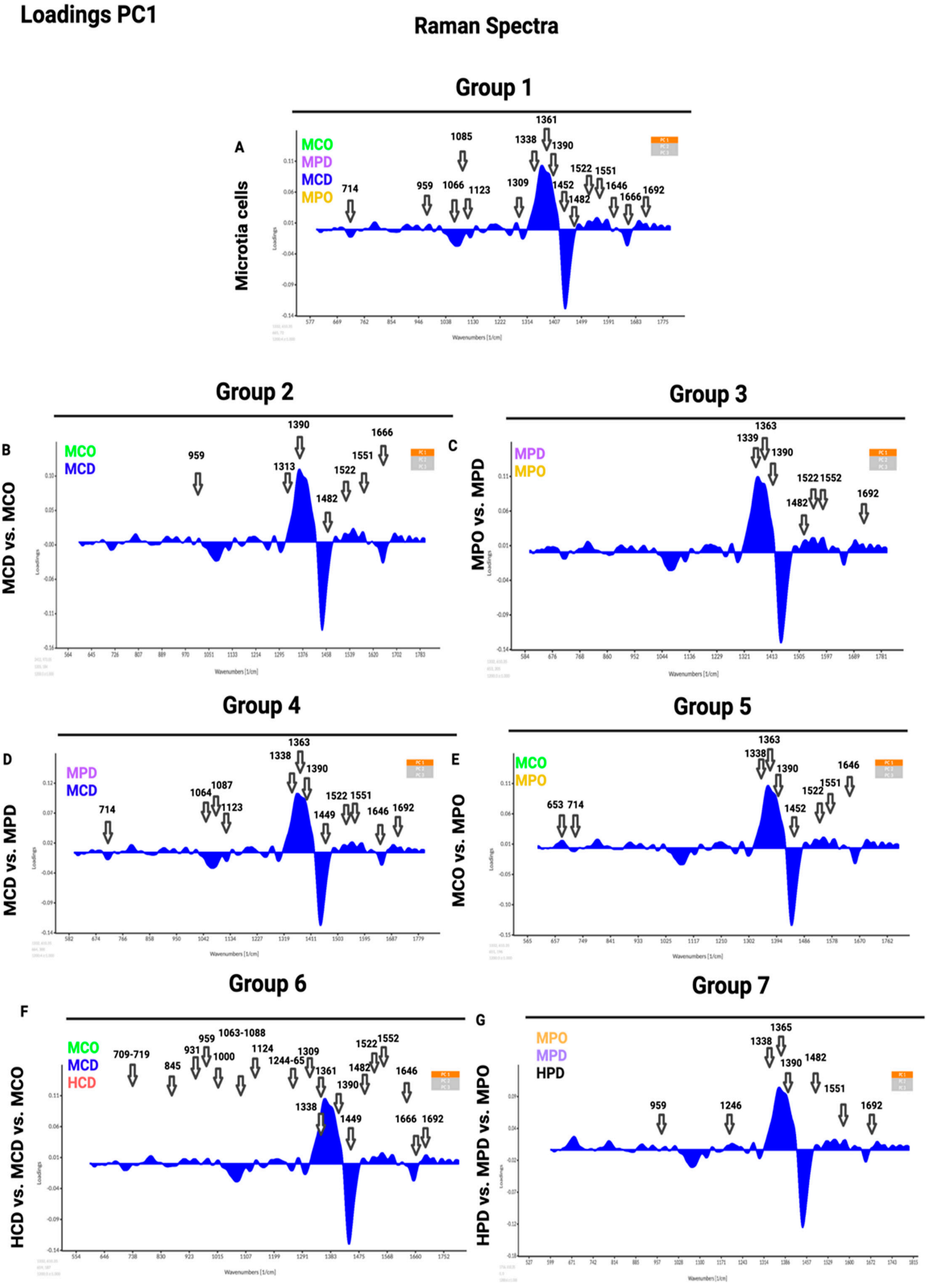
Loadings PC1
Raman Spectra
Group 1
A
Microtia cells
MCO
MPD
MCD
MPO
714
959
1066
1085
1123
1309
1338
1361
1390
1452
1482
1522
1551
1646
1666
1692
Wavenumbers [1/cm]
Group 2
B
MCD vs. MCO
MCO
MCD
959
1313
1390
1482
1522
1551
1666
Wavenumbers [1/cm]
Group 3
C
MPO vs. MPD
MPD
MPO
1339
1363
1390
1482
1522
1552
1692
Wavenumbers [1/cm]
Group 4
D
MCD vs. MPD
MPD
MCD
714
1064
1087
1123
1338
1363
1390
1449
1522
1551
1646
1692
Wavenumbers [1/cm]
Group 5
E
MCO vs. MPO
MCO
MPO
653
714
1338
1363
1390
1452
1522
1551
1646
Wavenumbers [1/cm]
Group 6
F
HCD vs. MCD vs. MCO
MCO
MCD
HCD
709-719
845
931
959
1000
1063-1088
1124
1244-65
1309
1338
1361
1390
1449
1482
1522
1552
1646
1666
1692
Wavenumbers [1/cm]
Group 7
G
HPD vs. MPD vs. MPO
MPO
MPD
HPD
959
1246
1338
1365
1390
1482
1551
1692
Wavenumbers [1/cm]

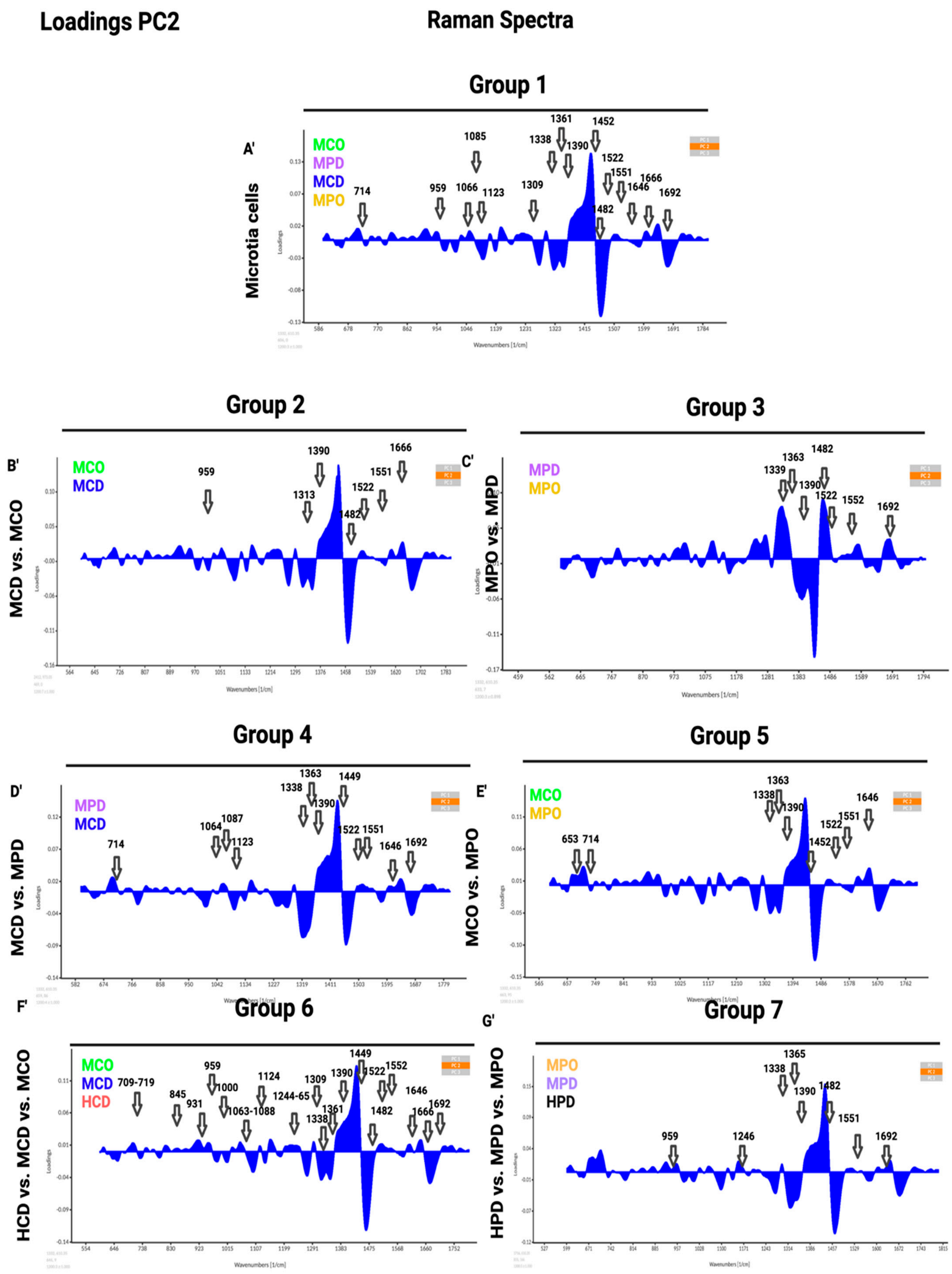
Loadings PC2
Raman Spectra
Group 1
A'
Microtia cells
MCO
MPD
MCD
MPO
714
959
1066
1085
1123
1309
1338
1361
1390
1452
1482
1522
1551
1646
1666
1692
Loadings
Wavenumbers [1/cm]
Group 2
B'
MCD vs. MCO
MCO
MCD
959
1313
1390
1482
1522
1551
1666
Group 3
C'
MPO vs. MPD
MPD
MPO
1339
1363
1390
1482
1522
1552
1692
Group 4
D'
MCD vs. MPD
MPD
MCD
714
1064
1087
1123
1338
1363
1390
1449
1522
1551
1646
1692
Group 5
E'
MCO vs. MPO
MCO
MPO
653
714
1338
1363
1390
1452
1522
1551
1646
Group 6
F'
HCD vs. MCD vs. MCO
MCO
MCD
HCD
709-719
845
931
959
1000
1063-1088
1124
1244-65
1309
1338
1361
1390
1449
1482
1522
1552
1646
1666
1692
Group 7
G'
HPD vs. MPD vs. MPO
MPO
MPD
HPD
959
1246
1338
1365
1390
1482
1551
1692

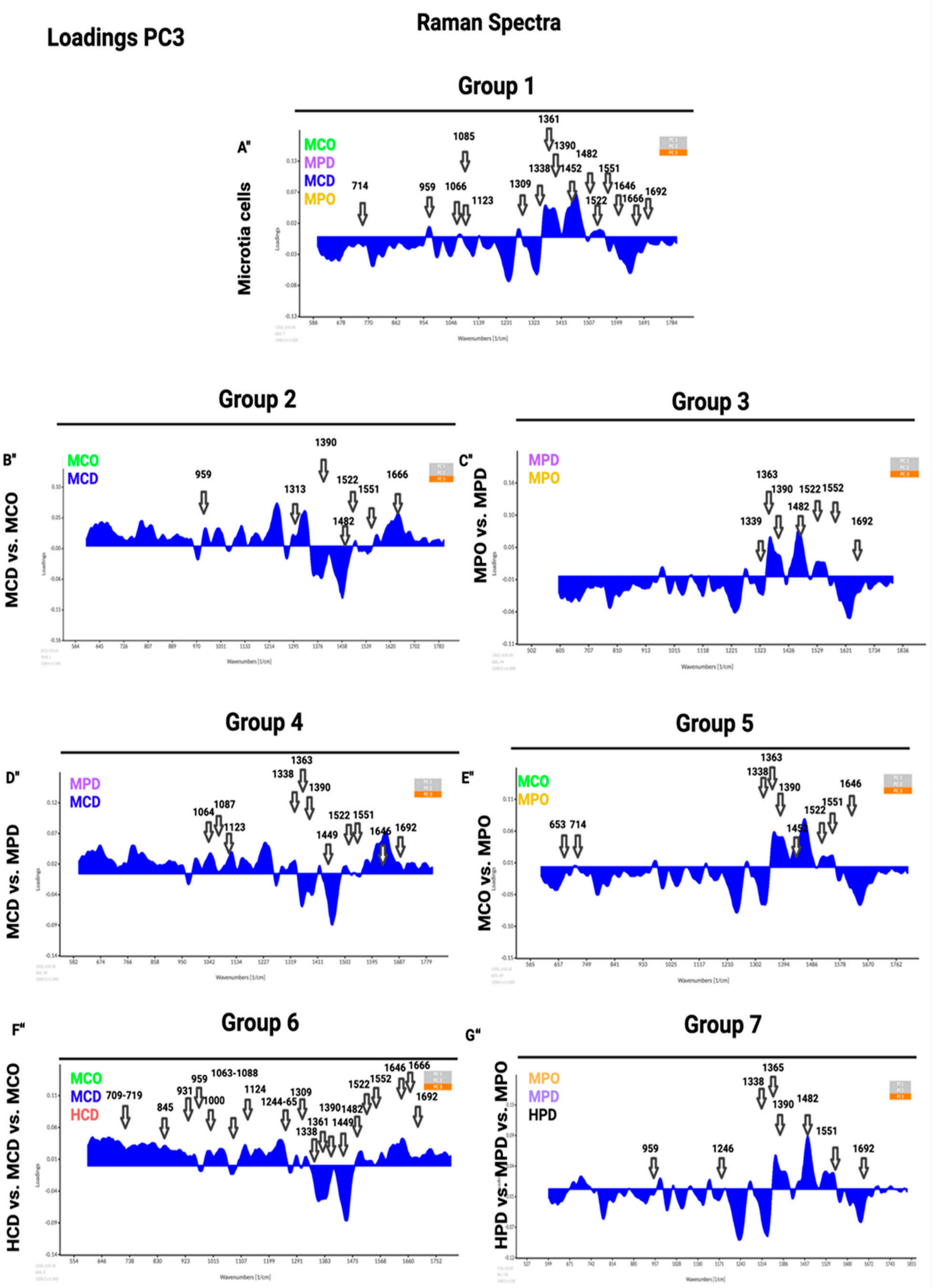

Figure S4. Visualization of Raman component (PC1/PC2/PC3) loadings plots of different cell populations. (**A**) PC1; **A'**, PC2; **A''**,PC3) Group 1: Comparison between MCD (n=133), MCO (n=177), MPD (n=179) and MPO (n=96), (**B**), PC1; **B'**, PC2; **B''**, PC3) Group 2: Comparison between MCD (n=133) vs. MCO (n=177), (**C**), PC1; **C'**, PC2; **C''**, PC3) Group 3: Comparison of MPD (n=179) vs. MPO (n=96), (D, PC1; D', PC2; D'', PC3) Group 4: Comparison between MCD (n=133) vs. MPD (n=176), (**E**), PC1; **E'**, PC2; **E''**, PC3) Group 5: Comparison between MCO (n=375) vs. MPO (n=220), (**F**), PC1; **F'**, PC2; **F''**, PC3) Group 6: Comparison between HCD (n=174) vs. MCD (n=133) vs. MCO (n=177), (**G**), PC1; **G'**, PC2; **G''**, PC3) Group 7: Comparison between HPD (n=140) vs. MPD (n=179) vs. MPO (n=96).

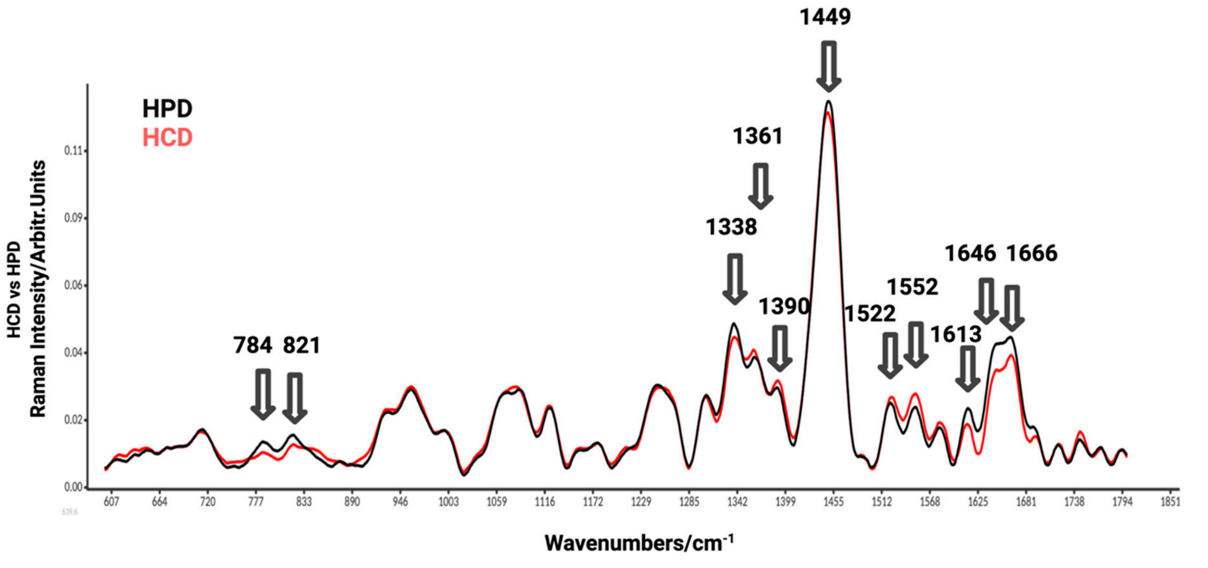

Figure S5: **Visualization of Raman mean spectra for healthy chondrocytes (HCD) (red) (n=174; n=6 donors) with healthy perichondrocytes HPD (black) (n=140; n=5 donors).**

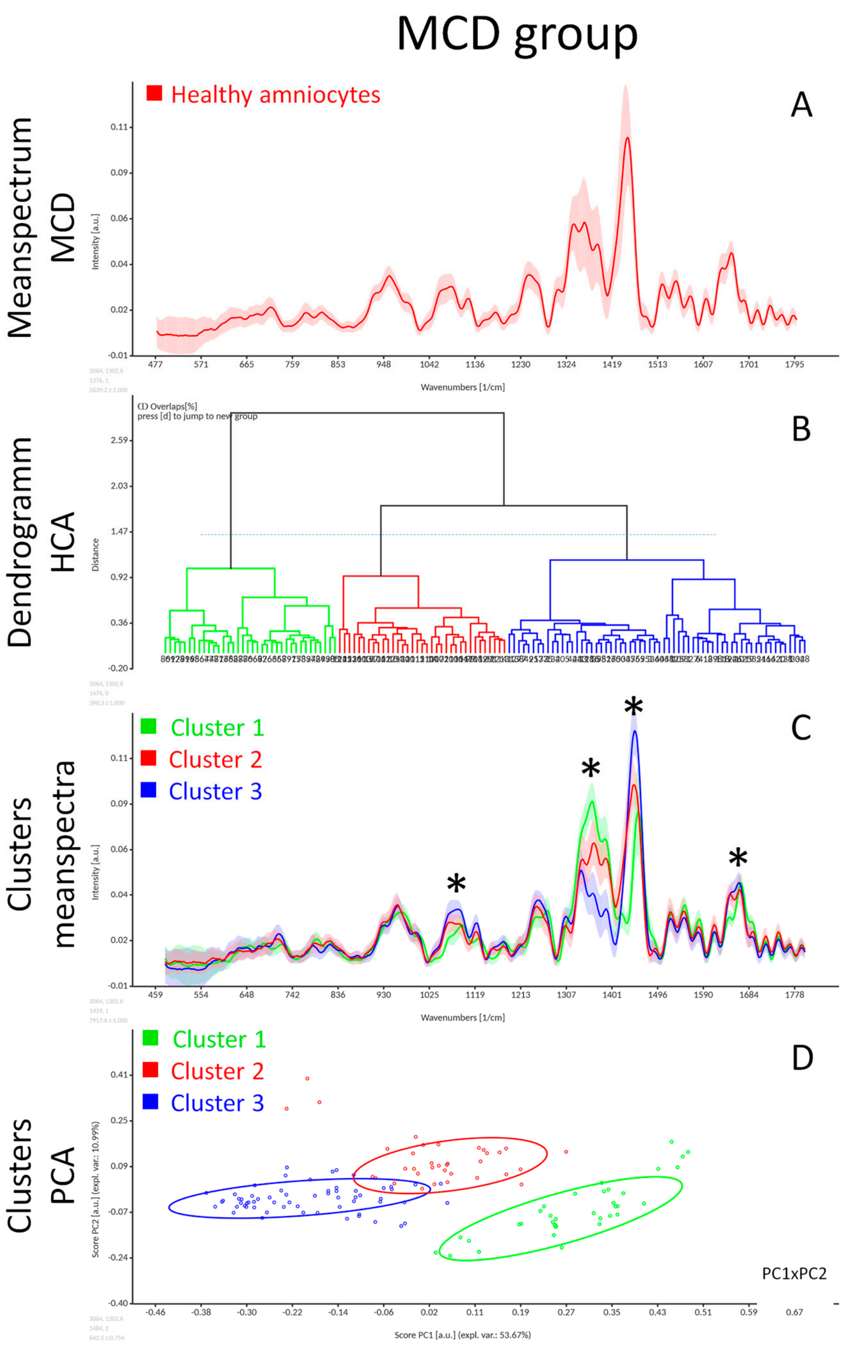

Figure S6. HCA and PCA of microtia chondrocytes. The Raman analysis of the merged MCD group is presented. (**A**) The mean spectrum of MCD group (4 donors, 133 spectra). Shadows represent the standard deviation. (**B**) HCA of the mean spectrum with the cut off shown indicated by the horizontal, dashed blue line. (**C**) Mean spectra of the clusters resulting from the HCA of Fig. S6B. The asterisks indicate the high variability in particular spectral regions. (**D**) PCA analysis with the PC1xPC2 score plot. Each dot represents a Raman measurement, and the ellipses describe 90% confidence interval.

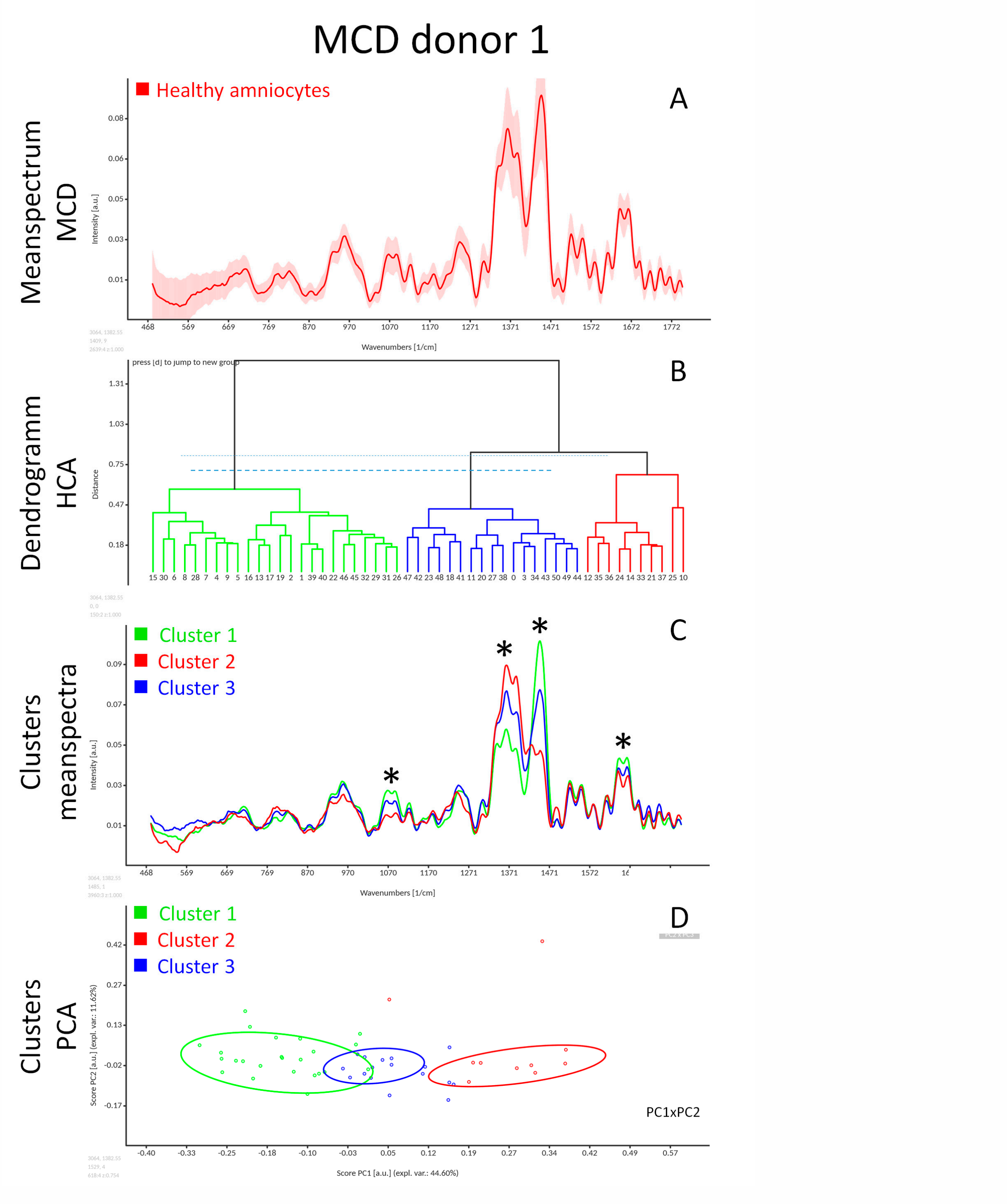

Figure S7. HCA and PCA of microtia chondrocytes. The Raman analysis of MCD donor 1 is presented. (**A**) The mean spectrum of MCD donor 1 (53 spectra). Shadows represent the standard deviation. (**B**) HCA of the mean spectrum with the cut off shown indicated by the horizontal, dashed blue line. (**C**) Mean spectra of the clusters resulting from the HCA of Fig. S7B. The asterisks indicate the high variability in particular spectral regions. (**D**) PCA analysis with the PC1xPC2 score plot. Each dot represents a Raman measurement, and the ellipses describe 90% confidence interval.

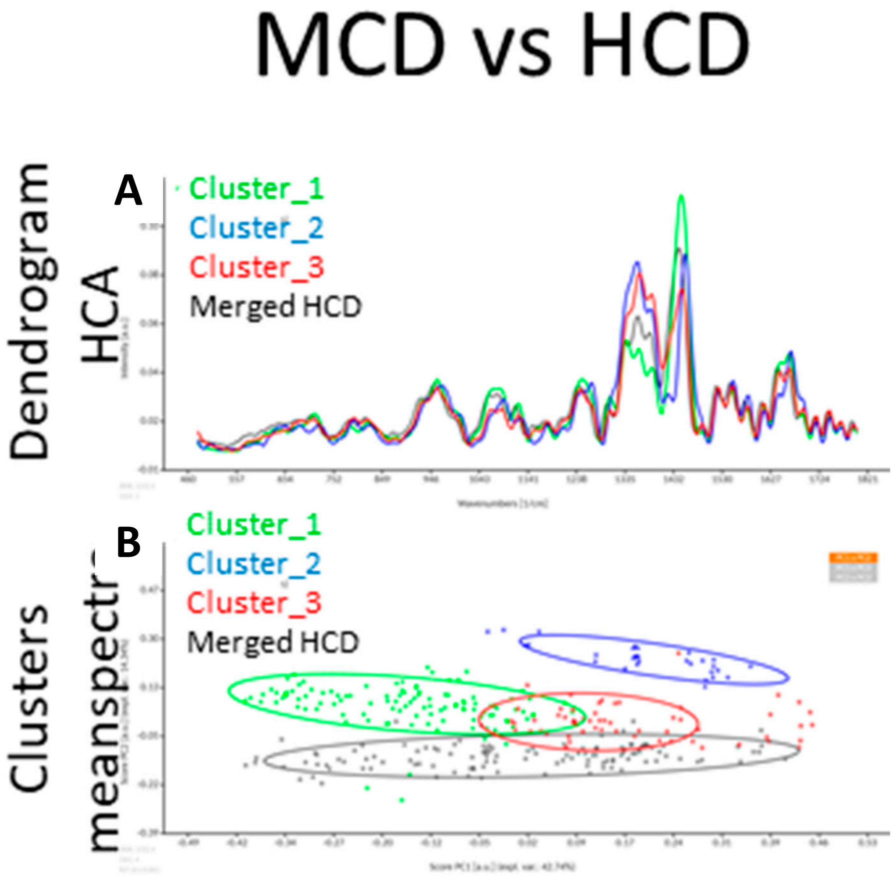

Figure S8. Comparison of putative MCD subpopulation with healthy chondrocytes. (**A**) Mean spectra of the three MCD clusters of Fig. S6 and the HCD population (Fig. 4A). (**B**) PCA analysis with the PC1xPC2 score plot. Each dot represents a Raman measurement, and the ellipses describe 90% confidence interval.

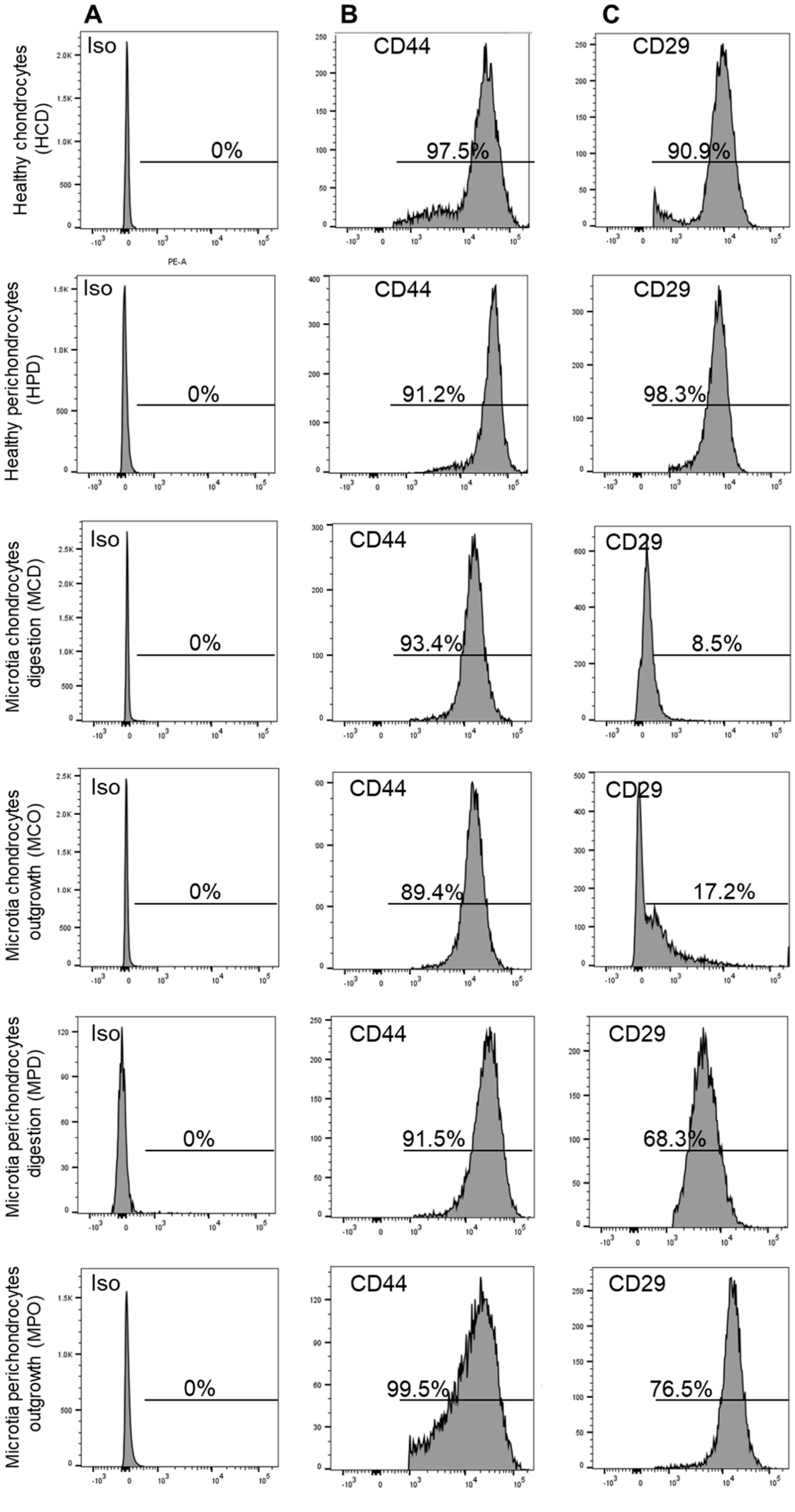

Figure S9 Flow cytometric analysis of the expression of CD44 and CD29 markers in healthy and microtia chondrocytes and perichondrocytes isolated by digestion or outgrowth. (**A**) Isotype controls (**B**) CD44 and (**C**) CD29 expression in cultured healthy (HCD) and microtia (MCD and MCO) chondrocytes as well as healthy (HPD) and microtia (MPD and MPO) perichondrocytes; all cells were used at passage 2–3; representative for $n = 5$ donors.
